# Successful pregnancy and live birth after intrauterine administration of autologous platelet-rich plasma in a woman with recurrent implantation failure: A case report

**Published:** 2017-12

**Authors:** Marzie Farimani, Jalal Poorolajal, Soghra Rabiee, Maryam Bahmanzadeh

**Affiliations:** 1 *Endometrium and Endometriosis Research Center, Hamadan University of Medical Sciences, Hamadan, Iran.*; 2 *Research Center for Health Sciences, Hamadan University of Medical sciences, Hamadan, Iran.*; 3 *Department of Epidemiology, School of Public Health, Hamadan University of Medical Sciences, Hamadan, Iran.*; 4 *Department of Anatomy, School of Medicine, Hamadan University of Medical Sciences, Hamadan, Iran.*

**Keywords:** Recurrent implantation failure, Platelet-rich plasma, Embryo transfer, Assisted reproductive technology, Pregnancy outcome, Embryo implantation

## Abstract

**Background:**

Platelets contain a significant amount of growth factors that have positive effects on local tissue repair and endometrial receptivity.

**Case:**

Here we present a 45-yr-old woman with primary infertility and two failed in vitro fertilization (IVF) cycles who was candidate to receive donor eggs. Five consecutive frozen-thawed embryo transfer cycles with good quality embryos were performed within 2 yr. With the diagnosis of recurrent implantation failure (RIF), the patient was treated for improving endometrial receptivity with intrauterine administration of autologous platelet-rich plasma (PRP), 24 hr before embryo transfer. The patient gave birth to a healthy baby boy weighing 2350 gr in the cesarean section.

**Conclusion:**

Extensive literature search suggests that this is the ﬁrst successful pregnancy after administration of PRP in a woman with RIF. Local administration of PRP before embryo transfer may play a vital role in successful implantation .

## Introduction

Despite progress has been made in the field of assisted reproductive technology, still multiple embryos fail to implant. A significant percentage of in vitro fertilization failure is due to the endometrial receptivity (1). Implantation requires a good quality embryo to provide a good coordination between mother and fetus. Various approaches have been used as therapeutic strategies in the investigations and management of RIF such as local endometrial injury, changes in stimulation protocols, intrauterine granulocyte colony-stimulating factor before embryo transfer, blastocyst assisted hatching transfer and pre-implantation genetic diagnosis for aneuploidy (2).

Human endometrium undergoes significant changes during implantation (3). Human endometrial tissue contains receptors for growth factors, adhesion molecules, cytokines, growth factors, lipids, and other factors are thought to enhance endometrial and embryonic development (4). PRP contains the growth factors and other cytokines that have positive effects on local tissue repair and endometrial receptivity. PRP could be a new treatment used for the improvement the endometrial thickness in women with a thin endometrium. The use of PRP is considered safe because it is autologous and is derived from patient’s own blood (5, 6).

Local administration of PRP could be one of the novels and probably successful treatment in women with recurrent implantation failure (RIF) (6-8). The successful outcome of pregnancy in a 45-yr-old woman with RIF is reported and discussed in this paper.

## Case report

The successful outcome of pregnancy in a 45-yr-old woman with RIF diagnosis received infertility treatment between 2013 and 2016 due to primary infertility that was reported. 

The primary routine infertility assessment and basal cycle day 3 serum levels of hormonal parameters were found to be normal (follicle-stimulating hormone: 7.4 mU/mL, luteinizing hormone: 4.3 mU/mL, and estradiol: 42.0 pg/mL). She described usually regular 26-day menstrual cycle with no history of gynecological disorders and no abnormal bleeding. As a result, intracytoplasmic sperm injection (ICSI) with controlled ovarian hyperstimulation was recommended. Two attempts of ovarian stimulation and embryo transfer were unsuccessful. Patient was diagnosed as a case of poor responder due to the production of less than five follicles after a standard stimulation protocol. Then she was counseled regarding her poor prognosis for conception and the option of ovum donation for ICSI.

During the first ovarian stimulation, 25 oocytes were obtained from a young donor woman and fertilized after conventional ICSI. Twenty good quality embryos were frozen at cleavage. The routine clinical approach to RIF investigation including uterine evaluation (hysteroscopy), thrombophilia testing (Lupus anticoagulant and anticardiolipin antibody) was normal.The third and fourth consecutive frozen-thawed embryo transfer cycles with good quality day 5 blastocysts were performed. 

Despite multiple embryo transfer, the patient remained committed to achieve pregnancy with the diagnosis of RIF. The couple was reconciled for poor outcome. Following informed consent, she underwent the frozen-thawed embryo transfer and intrauterine administration of autologous PRP. Blood samples were obtained from patient and PRP was prepared according to the standard protocol of the Iranian Blood Transfusion Organization (9). 0.5-1 mL PRP was infused to the uterine cavity under ultrasound guidance using Wallace catheter (Classic Embryo Replacement Catheter; Smiths Medical, Hythe, Kent, U.K) about 24 hr before undergoing frozen-thawed embryo transfer. Three embryos were transferred on the 5th day of progesterone administration.

Intramuscular progesterone (100 mg daily) for luteal support was provided.

on day 15 following embryo transfer, serum beta human chorionic gonadotropin was positive (>200 mIU/ml). Then transvaginal sonography was performed 15 days later to detect and confirm pregnancy and 2 wk later, clinical heart activity was observed.

The woman delivered a healthy baby boy weighing 2350 gr in the cesarean section in January 2017.

## Discussion

Implantation failure is a signiﬁcant challenge for clinicians and embryologists and is a major limiting step for assisted reproductive technology (10, 11). This is the ﬁrst report of a successful live birth from frozen-thawed embryo transfer in a woman with RIF and advanced reproductive age using intrauterine administration of autologous PRP. There are many pieces of evidence extensively demonstrated that PRP therapy is considered safe and promises many potential theoretical effects in different medical fields (6, 12, 13).

In 2015, Chang *et al* administered intrauterine infusion of PRP in infertile women with thin endometrium and reported 4 pregnancy from 5 patients with thin endometrium and poor response to conventional therapy during freezen embryo transfer (6). PRP contains the growth factors and other cytokines included transforming growth factor beta, fibroblast growth factor, platelet derived growth factor (PDGF), insulin-like growth factor I and II, vascular endothelial growth factor, epidermal growth factor (EGF), interleukin 8, keratinocyte growth factor, and connective tissue growth factor (14). 

Significant number of factors are known to exert paracrine influence on implantation stage endometrium (e.g., interleukin (IL)-1 beta, IL-6, IL-8, leukemia inhibitory factor (LIF), interferon gamma, and tumor necrosis factor) and support embryo implantation (e.g., chemokine ligand (CCL) 3, CCL4, CCL5, Fibroblast growth factor 2, granulocyte colony-stimulating factor, PDGF, tumor necrosis factor, and vascular endothelial growth factor) through their regulatory actions on proliferation, apoptosis, inflammation, cell adhesion, chemotaxis, and immune responses during blastocyst implantation (15).

Human endometrial tissue contains receptors for insulin-like growth factors, growth hormone, PDGF, EGF, and transforming growth factor beta isoforms. Many of these promote endometrial tissue remodeling and play a role by autocrine and paracrine, and are associated with endometrial receptivity, embryo implantation and development (16, 17). EGF regulates the production of vascular endothelial growth factor that locally contributes to decidual vascularization and placenta angiogenesis and endometrium proliferation, as well as playing key roles during successful embryonic implantation (18). 

However, we want to underline that in the case of implantation failure, PRP administration before embryo transfer may play a vital role in successful implantation and could be one of the novels and probably successful treatment in women with RIF. Embryo implantation requires complex interactions between blastocyst and endometrium including the interplay of endocrine and paracrine hormones, growth factors cytokines, and adhesion molecules. In the female reproductive tract, growth factors, cytokines, adhesion molecules, lipids, and other factors are thought to improve endometrial and embryonic development (4). Further studies are required to investigate the mechanisms responsible for the roles of PRP in endometrial receptivity in IVF cycles. Therefore, it is necessary to explore the precise effects of PRP on implantation.

## Conclusion

In our opinion this is the first report of successful pregnancy and live birth after intrauterine administration of autologous platelet-rich plasma in a woman with recurrent implantation failure. There is currently evidence to support the effectiveness of local administration of PRP in successful implantation. Further studies are needed to justify the benefits and clinical application of PRP in infertility treatment.
